# Correlates of mental disorders among minority Arab adolescents in Israel: results from the Galilee Study

**DOI:** 10.1186/s13584-018-0281-5

**Published:** 2019-01-21

**Authors:** Raida Daeem, Ivonne Mansbach-Kleinfeld, Ilana Farbstein, Robert Goodman, Rasha Elias, Anneke Ifrah, Gabriel Chodick, Rassem Khamaisi, Silvana Fennig, Alan Apter

**Affiliations:** 1 Sackler Faculty of Medicine, Tel Aviv University, Tel Aviv, Israel; 2 Child and Adolescent Mental Health Department, Ziv Medical Center, 13100 Zefat, Israel; 3 The Feinberg Child Study Center, Schneider Medical Center for Children in Israel, 49202 Petach Tikvah, Israel; 4 Department of Child and Adolescent Psychiatry, King College London Institute of Psychology, Psychiatry & Neuroscience, London, UK; 5 Israel Center for Disease Control, Gertner Institute, Sheba Medical Center, 5265601 Tel Hashomer, Israel; 6 School of Public Health, Tel Aviv University, Tel Aviv, Israel; 7 Epidemiology and Data Base, MaccabiTech, Tel Aviv, Israel; 8 Department of Geography and Environmental Studies, University of Haifa, 31905 Haifa, Israel; 9 Department of Psychiatry, Schneider Children’s Medical Center of Israel, 49202 Petach Tikva, Israel; 10 Ruppin Academic Center, Netanya, Israel; 11 Inter-Disciplinary Center, Herzliya, Israel

## Abstract

**Background:**

The Galilee Study is the first large epidemiological study to compare correlates of mental disorders between two Arab Palestinian minority groups of adolescents in Israel.

**Methods:**

A two-stage cross-sectional study, carried out between 2012 and 2014, included all 9th grade students from 5 Arab localities, representative of 77% of the Muslim and 100% of Druze citizens in Israel. During the screening stage, 1639 students completed the Strengths and Difficulties Questionnaire in the classroom (response rate = 69.3%). During the follow-up stage, 704 adolescent-mother dyads were interviewed at home; using the Development and Well-Being Assessment, the General Health Questionnaire (GHQ)-12, the Subjective Feeling of Discrimination Index (FDI), and socio-demographic questions (response rate = 84.4%).

**Results:**

Prevalence of any disorder, internalizing or externalizing disorders among Muslim adolescents were 19.2, 15.8 and 4.2%, respectively and among Druze adolescents 10.9, 5.9 and 5.5%, respectively. Muslim adolescents were 3.2 times more likely than Druze adolescents to have an internalizing disorder, while Druze were 2 times more likely than Muslim to have an externalizing disorder. Males were at higher risk than females for externalizing disorders in both populations, though among Druze the risk was more striking. Learning disabilities increased the likelihood of having an externalizing disorder in both populations. Risk factors for internalizing disorders among Muslim adolescents were female gender, a very low socio-economic level, few siblings, LD, high maternal GHQ-12 score and high FDI; and for externalizing disorders, male gender, a relatively low socio-economic level but not the lowest, learning disability and high maternal GHQ-12 score.

**Conclusions:**

We found an association between religion/ethnicity and internalizing and externalizing disorders as well as a strong correlation between religion/ethnicity and socio-economic variables. Therefore, we tend to conclude that not religion per se but the multifaceted socio-cultural and economic factors that characterize religious groups are associated with mental disorders. Very low socio-economic level and feeling discriminated which were traits connected only to Muslim adolescents, were associated with internalizing disorders. When preparing preventive measures aimed at furthering mental health among minority adolescents, authorities should focus on improving the socio-economic status of minorities and reducing institutional and personal discrimination. The educational and mental health establishments could undertake measures to improve resilience and coping strategies of Muslim families living in the most adverse conditions, such as providing special support through the school counseling services and coordinating, at the ministerial levels, school and community health services in order to carry out joint preventive programs and referrals to specialist services when needed.

## Introduction

Prevalence rates of any mental disorder in adolescents, according to worldwide community studies, range between 8.3 and 19.9% with a pooled prevalence of 13.4% [1]. Some studies have found higher rates of disorders in minority youth [2], while others have found lower rates [3].

Ethnic minorities are usually not homogenous and multiple inter and intra-group differences must be considered [4]. According to Adriaanse, Veling, Doreleijers & van Domburgh [5], inconsistent results related to whether minorities are better-off or worse-off than majority adolescents may depend on differing degrees of social disadvantage of the particular minority groups.

Risk factors that are particularly associated with belonging to a minority population include multiple stressors related to socio-economic disadvantage and adverse living circumstances, and limited access to health care. Daily encounters with discrimination is another risk factor for ethnic minorities [2], leading to lower self-esteem and social functioning [6]. Systematic reviews have found robust associations between perceived racial/ethnic discrimination and negative mental health outcomes, across different countries and cultures [7].

The Arab Palestinian citizens of Israel (Arabs in Israel) are an indigenous population, constituting about 18% of all Israeli citizens and 26.2% of those below 18 years of age. Over 80% of Arabs in Israel are Muslims, and the rest are mainly Druze and Christian. Ninety percent live in separated towns and villages [8]. Arabs in Israel are over-represented in all the indicators of poverty, distress and underdevelopment, with high unemployment rates and school drop-out rates [9]. In 2014, 63.5% of Arab children and adolescents lived below the poverty line compared with 21.6% of Jewish minors [10].

The Druze comprise a traditional and conservative Arabic-speaking cultural group [11] who participate in the Israeli military service. Druze men are usually employed in the security forces after completing their military service. This improves their economic status and increases their adoption of norms of the Jewish majority [12].

The Muslim Israeli citizens, on the other hand, are a non-assimilated minority, mainly due to the continuing state of conflict between Israel and the Arab world, which has placed them in the status of a hostile minority outside the national consensus [13].

The Israel Survey of Mental Health among Adolescents (ISMEHA) reported higher rates of internalizing and lower rates of externalizing disorders among Israeli Arab compared to Jewish adolescents [14]. The ISMEHA, however, assessed Israeli Arab adolescents as a single collective entity, possibly masking differences among the various subgroups [15].

The present study attempts to conduct a nuanced examination of the prevalence of mental disorders in two subgroups of adolescents comprising the Israeli Arab minority and to identify risk factors specific to these minority populations.

We hypothesized that: (1) Rates of psychopathology are higher among Muslim than among Druze adolescents; (2) Differences in rates of psychopathology are associated with economic factors rather than ethnic/religious factors.

## Methods

### The study population

The study included all 9th grade students in five localities, representative of the Muslim and Druze localities in northern Israel with regard to population size, geographic location and ethno-national composition. Not included in this study are Arabs living in mixed Jewish-Arab cities (10%) and in the southern Negev area (13%) [16]. Given that Muslims represent nearly 80% of the Arab minority in Israel and Druze only about 10%, we over-sampled for the Druze adolescents in order to have enough statistical power to be able to compare both populations. For this end, we selected two medium-sized Muslim towns and three smaller Druze towns. Regarding the age of the students, we chose to include older rather than younger adolescents, in order to be able to assess retrospective mental health history and difficult experiences. Ninth grade students were selected because the Compulsory Education Law of Israel determines that parents are obligated by law to send their children to school until 10th grade [17]. This choice would ensure that we would be able to interview the largest number of school attenders and follow them up to 10th grade before school drop-out rates increased.

### The sample

**Fig. 1:**
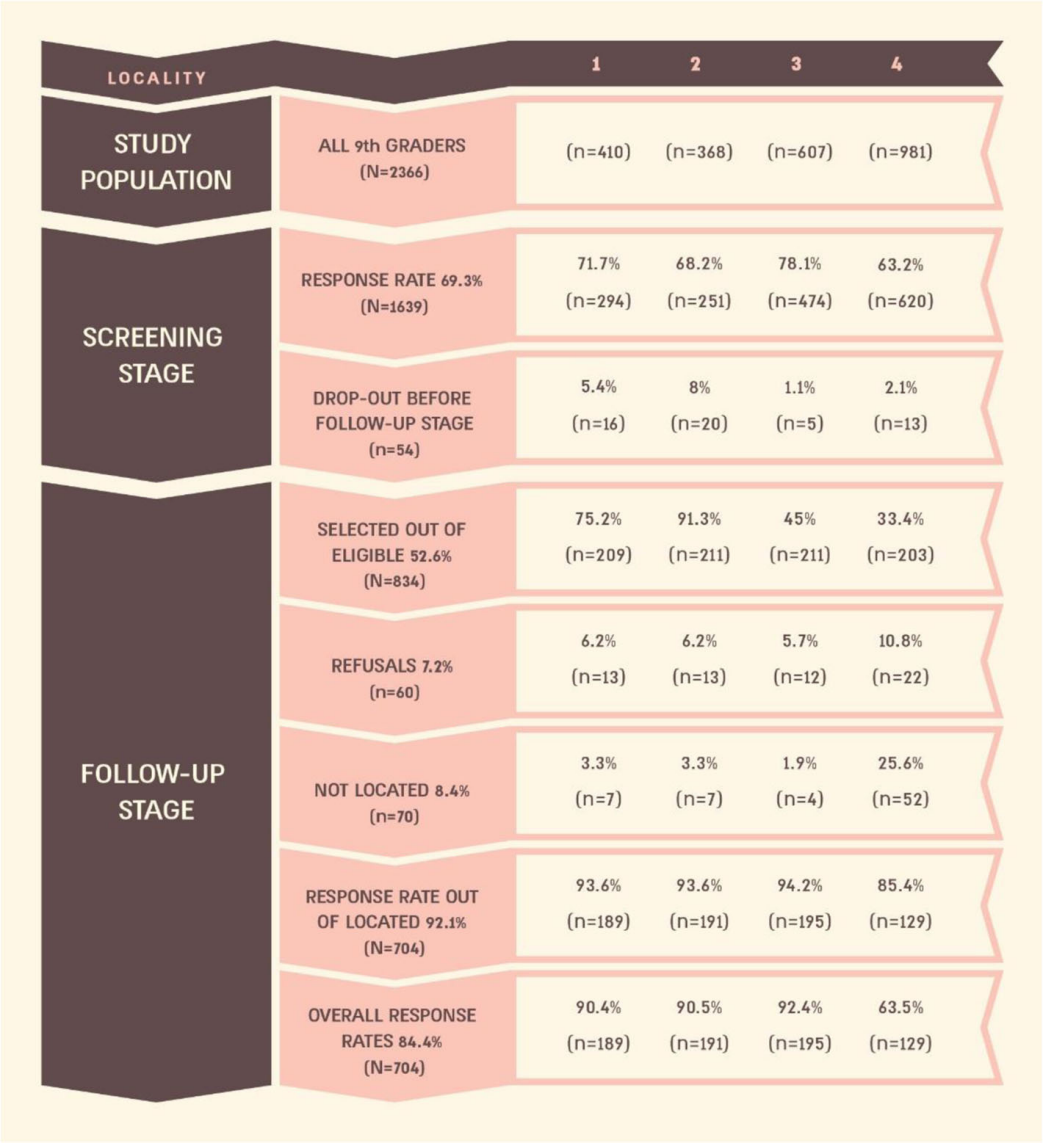
Results of the 2-stage data collection by locality: Numbers of participants and response rates

- *The sampling frame*: The sample included the 2012–2013 cohort of 9th grade students in the five localities (*N* = 2366). Not included were 220 adolescents who had dropped out, were non-school attenders, or were studying in out-of-town schools (see Fig. 1).
- *Sample size and sample probability*: The target was to reach approximately 1000 Muslim and 1000 Druze adolescents. The sample size was calculated as follows: The overall rate of mental disorders in Israel is 12% [14], and this rate could be obtained if we selected 39% of the quartile of adolescents scoring highest in the screening instrument and 3% of the remaining three quartiles. All 9th graders registered in school and attending class in these 5 localities were included in the study in order to reach the desired number of subjects for the initial screening stage (*N* = 2000), with an expected response rate of 70%. Data were analyzed after merging the two smaller Druze localities, very similar in size, ethnic composition and socio-economic characteristics, into one medium-sized locality (Locality 2).

### Instruments and measurements

- Emotional and behavioral problems were assessed with the self-report version of the Strengths and Difficulties Questionnaire (SDQ) – Arabic version (http://www.sdqinfo.org), a screening instrument designed for evaluating functioning in 4–17 year old children and adolescents [18]. It includes 25 items covering four clinical domains: hyperactivity-inattention, emotional symptoms, peer-relationship and conduct problems, and one pro-social behavior domain. The psychometric traits of the SDQ, have been examined in different cultural contexts and in clinical and epidemiological samples and have been found to be satisfactory [19]. The psychometric properties of the SDQ in Arabic have also shown to be satisfactory [20, 21].
- Mental disorders were assessed using the Development and Well-Being Assessment (DAWBA) – Arabic version – (https://www.dawba.info/b0.html), [20, 22], a multi-informant package of questionnaires, interviews and rating techniques that generate ICD-10 and DSM-IV psychiatric diagnoses for children aged 5–17. The category ‘internalizing’ disorders includes separation anxiety, specific phobias, social phobia, panic attacks and agoraphobia, post-traumatic stress, compulsion and obsessions, generalized anxiety and depression and deliberate self-harm, while the category externalizing disorders includes hyperactivity-inattention and awkward and troublesome behaviors and conduct disorders. In our study questions relating to troublesome behaviors (e.g., whether the child has lied, stolen or been questioned by the police), were censored by the Israeli Ministry of Education and excluded from the questionnaire, purportedly to prevent students’ self-incrimination. Computerized diagnoses combining responses of both adolescents and their mothers were generated based on the structured questionnaires. Subsequently, a team of experts confirmed or rejected the preliminary computerized diagnosis based on the comments recorded by the interviewers, providing a final clinical diagnosis for each adolescent. The initial validation study of the DAWBA showed its potential as an epidemiological measure and for clinic assessment and almost two decades of subsequent experience have confirmed this. “The DAWBA has been used in all the British nationwide surveys of child and adolescent mental health. These surveys, and similar surveys in many other countries, have generated reasonable prevalence rates, and have shown the expected pattern of association between disorders and independent risk factors - thereby providing further evidence for the validity of the DAWBA” [23].
- The socio-demographic data provided by the mothers included: religion; family size; parental educational; marital status; and whether the adolescent had a learning disability (LD).
- Socio-economic level of the locality was defined according to the ranking of local authorities published by the Central Bureau of Statistics of Israel [8]. The parameters used for this classification were: a) demography (median age, dependency rates and percentage of families with 4 or more children); b) education (average years of schooling of adults aged 25–54 and percentage with an academic degree); c) employment and benefits (percentage of salaried workers aged 15+, percentage of women aged 25–54 with no work-related income, percentage of employed earning double the average wage, percentage of workers earning less than minimum wage and percentage receiving benefits); and, d) standard of living (e.g. average monthly income and number of vehicles owned). The 255 Israeli local authorities are ranked and assigned to socio-economic clusters ranging from 1 (lowest) to 10 (highest). The localities included in this study belong to clusters 4, 3 and 2, as do nearly 90% of the Arab localities in Israel, and were classified into three socio-economic levels: a) medium (cluster 4), b) low (cluster 3), and c) very-low (cluster 2).
- General Health Questionnaire-12 item version (GHQ-12). The GHQ assesses mother’s risk of developing a psychiatric disorder as defined by distress and inability to carry out normal functions [24]. Mothers were categorized at high risk if they were in the highest 33% of the distribution and those in the lowest 67% were categorized at low risk. The Arabic version has an internal reliability (Cronbach alpha) of .86 [25].
- Subjective feelings of discrimination: Four items relating to how others respect one’s community, behave towards it, appreciate it and feel it contributes to the state were adapted from the Public Regard Subscale of the Multidimensional Inventory of Black Identity [26]. These four items were pooled into a “Feeling of Discrimination” Index (FDI) and categorized as high or low. The FDI had an internal reliability (Cronbach alpha) of 0.885.

### Study design

The study included a screening stage in the classroom and a follow-up stage at home. Adolescents with higher probability of having an emotional or behavioral problem according to the SDQ were over-sampled, in order to increase statistical power and the robustness of the analyses. In each locality all students in the highest 25% of the SDQ score distribution were considered at high risk for an emotional or behavioral problem and included in the sample for the follow-up stage, together with a simple systematic sample of students in the lower 75% of the distribution. The few Christian citizens or those from other denominations who lived in the relatively segregated Muslim or Druze towns and study in school were included in the screening stage, so not to exclude them or prevent their participation in the classroom when the rest of the students responded to the SDQ. However, they were not included in our comparative analyses.

### Procedures

Students whose parents signed an informed consent were recruited. They were asked to complete the SDQ in the classroom between September 2012 and May 2013. The follow-up took place between October 2013 and May 2014, when adolescents and their mothers were interviewed face-to face at their home, simultaneously and independently, by two lay interviewers. This study was approved by the Ethics Committee of the Rabin Medical Center (Request No, 6339).

#### Statistical analyses

Prevalence rates of internalizing and externalizing disorders were analyzed by socio-demographic and health-related risk factors, using Pearson Chi Square test, with a significance level set at ≤0.05. Multivariable binary logistic regressions were performed with internalizing or externalizing mental disorders as dependent variables. Logistic regression coefficients were transformed into odds ratios (OR) with 95% confidence intervals (CI). The adequacy of the model and goodness-of-fit were tested according to Hosmer and Lemeshow. All data were weighted to account for the sampling design in each locality and SDQ category, in order to generalize the study sample to the reference population (see Fig. 1) as follows: The inverse sampling probability of each individual in the sample was divided by the mean of the inverse sampling probabilities of all individuals in the group to yield a weighting variable scaled such that the mean weight of all individuals is 1 and the weighted sample size equals the actual, unweighted sample size [27]. Statistical analyses were conducted using an IBM SPSS-21 module (IBM Corp. Released 2012. IBM SPSS Statistics for Windows, Version 21.0. Armonk, NY: IBM Corp.).

#### Data collection and response rates

Figure 1 summarizes the two-stage data collection process. The cohort of 9th graders included 2366 students. During the screening stage, the highest response rate was achieved in Locality 3 (78.1%) and the lowest in Locality 4 (63.2%). The total response rate was 69.3%.

In the follow-up stage, each locality was assigned a different sampling fraction according to size, in order to include approximately the same number of subjects in each locality (e.g. 91.3% of the participants in the screening stage in Locality 2, and 33.4% in Locality 4). Response rates stood around 90%, except for Locality 4, where lack of street names and house numbers made finding the families difficult. Total response rate among located subjects was 92.3 and 84.5% when including refusals and not located in the “non-response” category.

Selected characteristics of adolescents who agreed to participate in the study (*N* = 1639), and of those who refused (*N* = 727), were compared. Among those who refused, there was a higher proportion of boys than among those who participated (59.4% vs. 42.5% respectively), and a higher proportion of students considered by teachers to be “low achievers” (34.7% vs. 21.9%, respectively). We found no differences between participants and non-participants by religion (Data not shown).

## Results

Prevalence of any disorder among Muslim and Druze adolescents was 19.2 and 10.9%, respectively (χ^2^ = 15.450; *p* = .000), while prevalence of internalizing disorders was 15.9 and 5.9% among Muslim and Druze adolescents, respectively (χ^2^ = 27.636; p = .000) and of externalizing disorders 4.2 and 5.5%, respectively (χ^2^ = 1.184; *p* = .277). Table 1 presents a description of the study population with the distribution of the selected risk factors (gender, socio-economic index, number of children in the family, family in welfare care, learning disability of the adolescent, GHQ-12 of mother and feelings of discrimination), and the prevalence of internalizing and externalizing mental disorders among Muslim and Druze adolescents by those risk factors.

**Table 1:** Prevalence of internalizing and externalizing mental disorders among Muslim and Druze adolescents by socio-demographic, psychosocial, health and school-related risk factors (weighted numbers and proportions)

Risk factors	Population group											
	Muslim *N* = 1147						Druze *N* = 440					
	N	%	Internalizing Disorder (15.8% *N* = 181)		Externalizing Disorder (4.2% *N* = 48)		N	%	Internalizing Disorder (5.9% *N* = 26)		Externalizing Disorder (5.5% *N* = 24)	
			%	(n)	%	(n)			%	(n)	%	(n)
Gender												
Male	551	48.0	13.2	(73)	6.2	(34)	211	48.0	5.2	(11)	10.8	(23)
Female	596	52.0	18.1	(108)	2.3	(14)	229	52.0	6.6	(15)	0.4	(1)
χ^2^; df; p			5.114; 1; .024		10.386; 1;,001				0.353; 1;.552		23.087; 1; .000	
SES Index												
Medium	-^a^						251	57.0	4.4	(11)	5.6	(14)
Medium-low	527	45.9	11.2	(59)	7.0	(37)	189	43.0	7.9	(15)	5.3	(10)
Very low	620	54.1	19.8	(123)	1.8	(11)	–					
χ^2^; df; p			15.941; 1; .001		19.609; 1; .000				2.449; 1; 118		.021; 1; .885	
Number of siblings												
1–2	287	25.0	27.5	(79)	2.1	(6)	126	28.7	8.7	(11)	2.4	(3)
3–4	569	49.6	14.9	(85)	5.3	(30)	237	53.9	4.2	(10)	6.8	(16)
5 or more	276	24.1	6.5	(18)	4.3	(12)	75	17.1	6.7	(5)	6.7	(5)
Missing	15	1.3					1	0.3				
χ^2^; df; p			47.229; 2; .001		4.767; 2; .092				3.084; 2; .214		3.280; 2; .194	
Family in welfare care												
Yes	268	23.3	14.2	(38)	5.4	(45)	67	15.3	4.5	(3)	7.5	(5)
No	838	73.1	16.9	(141)	1.1	(3)	365	83.0	5.8	(21)	5.2	(19)
Missing	42	3.6					8	1.7				
χ^2^; df; p			1.079; 1; .299		8.803; 1; .003				0.176; 1; 675		0.550; 1; .458	
Learning disability												
Yes	72	6.3	27.8	(20)	27.8	(20)	8	1.7	57.1	(4)	57.1	(4)
No	1065	92.8	15.2	(161)	2.6	(28)	432	98.3	4.9	(21)	4.4	(15)
Missing	11	0.9										
χ^2^; df; p			7.979; 1; .005		105.141; 1; .000				35.060; 1; 000		38.600; 1; .000	
GHQ-12 of mother												
High	414	36.1	22.9	(95)	7.5	(31)	100	22.8	4.7	(16)	9.0	(9)
Low	673	58.6	12.6	(85)	1.9	(13)	340	77.2	10.0	(10)	4.4	(15)
Missing	60	5.3										
χ^2^; df; p			19.648; 1; .000		20.374; 1; .000				3.895; 1; 048		3.154; 1; .076	
FDI^b^												
High	353	30.7	20.1	(71)	5.4	(19)	7	1.7	0		25.0	(2)
Low	720	62.8	12.5	(90)	3.6	(26)	427	97.1	6.1	(26)	5.2	(22)
Missing	75	6.5					5	1.2				
χ^2^; df; p			10.765; 1; .001		1.877; 1; .171				.452; 1; .501		5.934; 1; .015	

+ *p* ≤ .05; ++ *p* ≤ .01; +++ *p* ≤ .001; ^a^ ‘-’ = no cases in this category; ^b^ Feelings of Discrimination Index

Bi-variate analyzes on Table 1 show an association between gender and internalizing disorders only among Muslim adolescents, with females having higher rates than males. Regarding externalizing disorders, both Muslim and Druze males had higher rates of externalizing disorders than females. Regarding socio-economic index, a complex association was found among Muslim adolescents only: a very low socio-economic level was associated with a very high prevalence of internalizing disorders (19.8%) and with a very low prevalence of externalizing disorders (1.8%). Regarding number of siblings, fewer siblings were associated with higher rates of internalizing disorders among Muslim adolescents only. A high maternal GHQ score was associated with higher rates of internalizing disorders among both Muslim and Druze adolescents and with externalizing disorders among Muslim adolescents only. Regarding feelings of discrimination, a high score was associated with higher rates of internalizing disorders among Muslim adolescents only. Number of Druze adolescents with high FDI were few. Learning disability was associated with higher rates of internalizing and externalizing disorders in both Muslim and Druze groups, though number of Druze adolescents with a LD were few.

Table 2 shows the results of logistic regression analyses performed to assess the risk associated with internalizing and externalizing disorders among Muslim and Druze adolescents separately. The independent variables included were those that showed a strong association with internalizing and externalizing disorders in the bi-variate analyses, and showed also a sufficient number of subjects in each cell.

**Table 2:** Internalizing and externalizing mental disorder in Muslim and Druze adolescents by selected socio-economic, psychosocial and health-related factors: logistic regression (OR and 95% CI)

Risk factors	Population group			
	Muslim *N* = 1147		Druze *N* = 440	
	Internalizing Disorder (*N* = 181–15.8%)	Externalizing Disorder (*N* = 48–4.2%)	Internalizing Disorder (*N* = 26–5.9%)	Externalizing Disorder (*N* = 24–5.5%)
	OR (95% CI)		OR (95% CI)	
Gender				
Male	1.00 [reference]	3.2 (1.3–7.5)++	1.00 [reference]	24.6 (3.8–161.3) +++
Female	1.5 (1.0–2.1) +	1.00[reference]	1.5 (0.6–3.3)	1.00 [reference]
SES Index				
Medium	------^a^	–	1.00 [reference]	1.8 (0.7–4.7)
Medium-low	1.00 [reference]	5.0 (2.0–8.8)+++	1.5 (0.6–3.5)	1.00 [reference]
Very low	2.4 (1.6–2.8)+++	1.00 [reference]	–	–
Number of siblings				
1–2	6.3 (3.3–11.8) +++	1.00 [reference]	1.7 (0.5–5.7)	1.00 [reference]
3–4	3.2 (1.8–5.7)+++	2.8 (0.9–6.5)+	0.8 (0.3–2.5)	3.6 (0.9–13.4)
5 or more	1.00 [reference]	1.7 (0.5–6.5)	1.00 [reference]	3.5 (0.7–16.8)
Learning disability				
Yes	4.0 (2.1–7.5)++	21.8 (8.9–53.4) +++	–	–
No	1.00 [reference]	1.00 [reference]		
GHQ-12				
High	2.0 (1.4–2.9)+++	8.7 (3.8–19.5)+++	0.5 (0.2–1.1)	2.0 (0.7–5.4)
Low	1.00 [reference]	1.00 [reference]	1.00 [reference]	1.00 [reference]
FDI Index			–	–
High	2.5 (1.7–3.6)+++	1.6 (0.7–3.5)		
Low	1.00 [reference]	1.00 [reference]		

+ p ≤ .05; ++ *p* ≤ .01; +++ *p* ≤ .001

^a^ ‘------’ = less than 5 cases in this category

Table 2 shows that among Muslim adolescents, females were 1.5 times more likely than males to have an internalizing disorder. Those in the lowest socio-economic index were 2.4 times more likely to have an internalizing disorder than those in the low socio-economic index. Adolescents who had 1 or 2 siblings and those who had 3–4 siblings were 6.3 and 3.2 times more likely, respectively, than those who had 5 or more siblings to have an internalizing disorder. Adolescents with a learning disability were 4 times more likely than those without to have an internalizing disorder and those whose mother had a high GHQ score were 2 times more likely than those whose mother had a low GHQ score to have an internalizing disorder. Adolescents with high FDI score were 2.5 times more likely than those with a low FDI score to have an internalizing disorder.

The likelihood of having an externalizing disorder among Muslim adolescents, over and above the effect of the other variables, was as follows: males were 3.2 times more likely than females to have an externalizing disorder. Adolescents in the low socio economic index were 5 times more likely to have an externalizing disorder than those in the lowest socio-economic index. Adolescents with a learning disability were 21.8 times more likely than those without to have an externalizing disorder and those whose mother had a high GHQ score were 8.7 times more likely than those whose mother had a low GHQ score to have an externalizing disorder.

Table 2 shows that among Druze adolescents, when included in a multivariate analysis none of the independent variables examined were associated with a higher risk of having an internalizing disorder over and above the effect of the other variables, although in the bi-variate analyses LD and GHQ of the mother were found to be risk factors. Regarding externalizing disorders, LD and FDI were excluded from the regression analysis due to very small number of cases. We found that only gender represented a strong risk factor with males being 24.6 times more likely than females to have an externalizing disorder, over and above the effect of the other variables.

In this sample religion was highly correlated with socio-economic index (Pearson correlation = .707). Therefore, multivariate analyses were carried out to assess the association between mental disorders and religion over, and above the effect of the other variables, without the desired socio-economic markers. Within this limitation, Table 3 shows that religion was associated with both internalizing and externalizing disorders, over and above the effect of gender, number of siblings, learning disability, whether or not the family is in welfare care and maternal GHQ scores. Muslim adolescents were 3.2 times more likely to have an internalizing disorder, while Druze adolescents were 2 times more likely to have an externalizing disorder.

**Table 3:** Internalizing and externalizing mental disorders in minority adolescents by socio-economic, psychosocial and school related factors: logistic regression analysis (OR and 95% CI)

Risk factors	Internalizing disorder OR (95% CI)	Externalizing disorder OR (95% CI)
Gender		
Male	1.00 [reference]	5.6 (2.8–11.0) +++
Female	1.6 (1.2–2.2) +++	1.00 [reference]
Religion		
Muslim	3.2 (2.0–5.0)+++	1.0 [reference]
Druze	1.00 [reference]	2.0 (1.1–3.6) ++
In welfare care		
Yes	0.9 (0.6–1.4)	2.7 (1.2–6.0)++
No	1.00 [reference]	1.00 [reference]
Number of siblings		
1–2	7.7 (4.5–13.3) +++	1.00 [reference]
3–4	3.1 (1.8–5.2) +++	3.8 (1.6–8.9)] ++
5 or more	1.00 [reference]	2.1 (0.8–5.7)
Learning disability		
Yes	5.2 (2.9–9.4)+++	20.8 (10.4–41.6) +++
No	1.00 [reference]	1.00 [reference]
GHQ-12		
High	2.4 (1.7–3.3)+++	4.3 (247.7) +++
Low	1.00 [reference]	1.00 [reference]

+ *p* ≤ .05; ++ *p* ≤ .01; +++ *p* ≤ .001

R-squared values ranged between .15 and .39.

## Discussion

The Galilee Study had as its goal to examine separately Muslim and Druze adolescents, which the ISMEHA study carried out in 2004–5 [14] had included as one single entity, in order to assess two questions. One, whether ethnicity/religion had a particular impact on prevalence of mental disorders, over and above the effect of other variables, mainly social disadvantage. The other, whether mental disorders were associated with different risk factors among Muslim and Druze adolescents. The fact that these different ethnic/religious groups reside in relatively segregated communities, have different degrees of neighborhood disadvantage and varying perceptions of suffering from discrimination by the majority population, offers a unique opportunity to examine underlying socio-economic factors that may be related to specific mental disorders in minority populations.

Regarding our first question, our findings seem reflected in Dogra’s et al., [4], statement that there is “a complex interplay between minority status and social class, with terms such as ethnicity being a proxy for multifaceted sociocultural and economic variables” (p. 265). We did find a higher prevalence of internalizing disorders among Muslim than among Druze adolescents. However, we cannot explain this finding without taking into consideration the socio-economic disadvantage of the Muslim population as compared to the Druze population or, in the words of Dogra et al. [4], the multifaceted sociocultural and economic variables that characterize the distinct ethnic/religious groups. Therefore, we cannot arrive at unreserved conclusions regarding the association between mental health and religion per se, as religion is not a discrete variable isolated from other socio-cultural and economic factors. In our study population, religion was associated with several measures of disadvantage. Muslim adolescents were in the low and lowest socio-economic levels, as compared to Druze who were in the medium and low levels. Muslim families had higher rates of welfare care than Druze families, had higher rates of adolescents with a learning disability, higher rates of maternal GHQ morbidity and much higher rates regarding feelings of discrimination than Druze adolescents. These factors present a picture of poverty and family stress, as exemplified by the fact that 23.3% of Muslim families were in welfare care, as compared to 15.3% of Druze families and by the fact that in 36.1% of Muslim families the mother had a high GHQ score, as compared to 22.8% among Druze families. Other socio-cultural factors that address the challenges facing adolescents belonging to minority groups in a disadvantaged social position are feelings of discrimination. In the case of Israel, decreed by law as a Jewish and democratic state (Basic Law: Human Dignity and Liberty, 17.3.1992), belonging to a religion/ethnicity other than Jewish brings additional discrimination and disadvantage. We also found in this respect a great disparity between Muslim and Druze adolescents, with 30.7% of Muslim declaring they feel discriminated as compared to 1.7% among Druze. Feeling discriminated against by the mainstream population and the negative impact of cultural or political mistrust are important factors affecting mental health, depression, conduct problems and well-being among minorities [6, 7, 28].

Regarding our second question, whether risk factors for internalizing and externalizing disorders differ between these two minority groups, we found marked variations that reflect the different socio-economic conditions between Muslim and Druze and their different relation vis-à-vis the dominant Jewish population. The risk factors for internalizing disorders among the Muslim adolescents were female gender, a very-low socio-economic index, having few siblings, having a learning disability, a high maternal GHQ score and feeling very discriminated. Regarding externalizing disorders (ADHD and ODD), the risk factors for Muslim adolescents were being male, having a medium socio-economic index, having 3–4 siblings having a learning disability and a mother with a high GHQ score. In contrast, these risk factors were found to be not significantly associated with internalizing disorders among Druze adolescents, over and above the effect of each other, while only gender was found to be a strong risk factor for externalizing disorders, with males being 24.6 times more likely than females to have an externalizing disorder.

Our study confirmed the finding of others [14, 29–31], regarding higher prevalence of internalizing disorders among females and higher prevalence of externalizing disorders among males. As well, our findings confirm what others have revealed, that socio-economic factors associated with ethnicity are most likely to explain mental health problems in ethnic minorities [5, 32, 33]. The socio-economic index used in this study [8], showed that nearly all Arab local authorities (*N* = 84), were ranked below the Israeli average and allowed to distinguish among different degrees of disadvantage, i.e., among the medium, low and very-low SES groups. Like McLeod [34], we found more internalizing disorders with decreasing socio-economic level among Muslim adolescents: those in the very- low socio-economic level were 2.4 times more likely to have an internalizing disorder than those in the level immediately above theirs. Langton, Collishaw, Goodman, Pickles & Maughan [35], claim that the gap between the very-low income group and the rest has widened and that the increase in relative inequality “might lead to a disproportionate increase in emotional problems in low-income groups” (p. 1086). This they attribute to the possibility that poorer families are exposed to more risk factors for emotional problems, such as loss of self-esteem and sense of control, and the possibility that factors associated with low income have become “more powerful risks for emotional difficulties over time” (p. 1086). Ford, Goodman and Meltzer [36] also found a greater gap between the very low socio-economic levels and those a slightly above them regarding exposure to adverse life events and maternal distress and family dysfunction. It is likely that among the Druze adolescents, who were all either in the medium or low socio-economic levels, the gap was less meaningful and the two groups were more homogenous than the Muslim adolescents who were either in the low or very-low socio-economic levels, where this gap was more significant. This would explain why among the Druze, internalizing and externalizing disorders in the bi-variate analyses showed an association with the youth’s particular characteristics (gender, LD and maternal GHQ score) rather than with measures of socio-economic level and neighborhood disadvantage.

Brody and colleagues [33] address the important contribution of economic hardship, neighborhood poverty and racial discrimination as risk factors associated with children’s and adolescents’ mental health. They acknowledge, though, that family- centered programs can provide health benefits by improving the resilience of those families living in the increased adversity of poverty and discrimination. Thiede and colleges [32] show that persistent and increasing racial inequality among Hispanic and Black Americans, compared with the White majority, bring America closer to a majority- minority society. They assess the multiple factors underlying race differences, such as having been born into poor families, being over-represented in high poverty areas and having less contact with social safety nets- all as factors associated with higher risk for mental health problems.

Over 30% of Muslim adolescents felt discriminated against by the majority, as opposed to 1.7% among the Druze. It seems that the participation of the Druze in the Israeli military and security services has given them a stronger sense of being appreciated and of being perceived as contributing to the well-being of the state of Israel, as compared with the Muslim citizens. The claim of Frantz Fanon [37] that dominant groups tend to implant their hegemony by inculcating an image of inferiority – a depreciating self-image - in the subjugated, is of particular relevance when dealing with the relations between the Jewish majority and the institutionally discriminated Muslim Israeli minority.

Regarding externalizing disorders, however, we found different trends: Muslim adolescents in the low socio-economic level were 5 times more likely to have an externalizing disorder than those in the very-low level. This finding may be explained by the excess of ADHD cases in the externalizing category and the socio-economic differential that exists in ADHD diagnoses in Israel where underdiagnoses in minorities might be very much influenced by lower socio-economic level [38]. The ISMEHA showed that in the total Israeli population, lower SES was less likely to be associated with ADHD [39]. Other studies, however, present different results and claim that ADHD is more prevalent among the more disadvantaged population [40].

We found, like Parry Langdon [41], higher rates of internalizing disorders among adolescents with fewer siblings: those with 1–2 siblings or 3–4 siblings were 7.7 and 3.1 times more likely, respectively, than those with 5 or more siblings to have an internalizing disorder. A possible link to our findings is the observation that in single-child families, children often report feelings of loneliness, boredom and inferiority [42]. Our finding, however, seems to be particular to the Israeli traditional minorities and seems not to reflect what occurs in other populations [36].

Regarding externalizing disorders, we found the opposite trend: adolescents in medium- sized families (3–4 siblings) were 3.8 times more likely than smaller families (1–2 siblings) to have an externalizing disorder. These findings may be explained by the fact that in our study almost 60% of medium-sized families were in the higher socio-economic index, where we find more diagnosed ADHD cases.

Comparing adolescents with LD and those without we found, like Prior et al., [43], a higher prevalence of internalizing disorders (5.2 times more likely) among adolescents with a LD and a much higher prevalence of externalizing disorders (20.8 times more likely), probably due to the co-occurrence of LD and ADHD.

Adolescents whose mothers had a high-risk GHQ score were 4.3 times more likely to have an externalizing disorder and 2.4 times more likely to have an internalizing disorder than those whose mothers had a low risk score. Gonzales et al., [44], describe how multiple overlapping contextual influences relevant to low-income status, like economic hardship and neighborhood disadvantage, operate together to shape parenting and ultimately affect adolescent mental health.

### Limitations

One of the limitations of the Galilee Study is that the sample includes only students and therefore missed school dropouts or non-attenders who may be suffering from physical or mental problems. However, we estimate that this group is well represented because school attendance in Israel is mandatory for the age group examined. Dropout rates in Localities 1, 2 and 3 were below 2%, and in 8% in Locality 4. Additionally, we found higher refusal rates among males than among females, and this may have underrepresented the prevalence of externalizing disorders, which are more common among males. Refusal rates were also higher among students that were assessed by teachers as having low achievement and, therefore, our results might be under-representing the most severe cases. An unfortunate limitation, which we could not overcome, was that the Israeli Ministry of Education censored and excluded questions related to conduct disorders from the questionnaire. Therefore, externalizing problems in our study lack the conduct component and might be under-represented. Lastly, it is important to stress that we over-sampled for the Druze adolescents in order to match their numbers to those of the Muslim adolescents and thus obtain enough statistical power to compare two representative groups.

Although our R-squared values are somewhat low, this is usual when attempting to predict human behavior. However, we have statistically significant predictors and therefore we can draw important conclusions about how changes in the predictor values are associated with changes in the response value.

## Conclusions

Our major findings stress the importance of socio-economic variables, rather than religious/ethnicity configurations, regarding adolescent psychopathology. An important finding, which needs further examination, is that adolescents in the lowest socio-economic level are at much higher risk for internalizing mental disorders than those with somewhat better socio-economic conditions. Our findings lead us to conclude that attributing mental health problems to religious affiliation alone is a mistake and that the focus of the establishment needs to shift toward changing the socio-economic conditions of minorities in the lowest socio-economic ranking and towards prevention of institutional and personal discrimination.

Before these desired structural social changes take place, however, there are measures that could be undertaken by the educational and mental health establishments to improve resilience and coping strategies of Muslim adolescents and their families living in the most adverse conditions. Schools in these localities should be prepared to provide support by increasing their counseling activities to adolescents and their families. As well, the HMOs serving these localities should be aware of the specific needs of adolescents and their families and be able to identify those in distress when preventive measures can still be employed and to refer those who need it to specialist services. Coordination between school services and community services provided by the mental health clinics for children and adolescents at the HMOs are warranted and efforts to achieve this should be carried out at the ministerial levels.

Regarding feelings of discrimination, efforts should be made to influence the judicial and legislative branches of government to enact antidiscrimination laws and protect minorities in Israel.

These findings refer to the indigenous population of Israeli Muslim and Druze citizens living in a Jewish State but have far-reaching implications for other countries where religious/ethnic variability exists.

## Abbreviations

ADHD: Attention deficit hyperactive disorder
DAWBA: The development and well-being assessment
FDI: Feeling discrimination index
GHQ: General Health Questionnaire
ISMEHA: Israel Survey of Mental Health among Adolescents
LD: Learning disability
ODD: Oppositional deficit disorder
SDQ: Strengths and Difficulties Questionnaire
